# Pulmonary Vein Thrombosis Associated with Metastatic Follicular Thyroid Carcinoma: A Case Report and Review

**DOI:** 10.1155/2018/6096704

**Published:** 2018-07-19

**Authors:** Maria Mavromati, Annick Gerber, Gregor John

**Affiliations:** ^1^Department of Internal Medicine, Rehabilitation and Geriatrics, Geneva University Hospitals (HUG), Gabrielle-Perret-Gentil 4, 1205 Geneva, Switzerland; ^2^Department of Internal Medicine, Hôpital Neuchâtelois, 2300 La Chaux-de-Fonds, Switzerland

## Abstract

Pulmonary vein thrombosis (PVT) mainly occurs following lung transplantation but cases associated with thoracic malignancy have also been described. We describe here the first case of PVT in an asymptomatic patient with metastatic follicular thyroid carcinoma.

## 1. Introduction

Pulmonary vein thrombosis (PVT) is a rare condition [1]. Most cases described in medical literature occurred after thoracic surgery [2–4], especially lung transplantation [5]. PVT has also been associated with thoracic malignancy [4–6] and central catheter placement [7], but idiopathic cases have sporadically been described [1, 8]. Diagnosis is challenging due to its nonspecific symptoms, with most cases being incidentally diagnosed during thoracic imaging. We describe here the first case of pulmonary vein thrombosis associated with metastatic thyroid carcinoma in an asymptomatic patient and review current diagnostic and management strategies.

## 2. Case Summary

**Figure 1:**
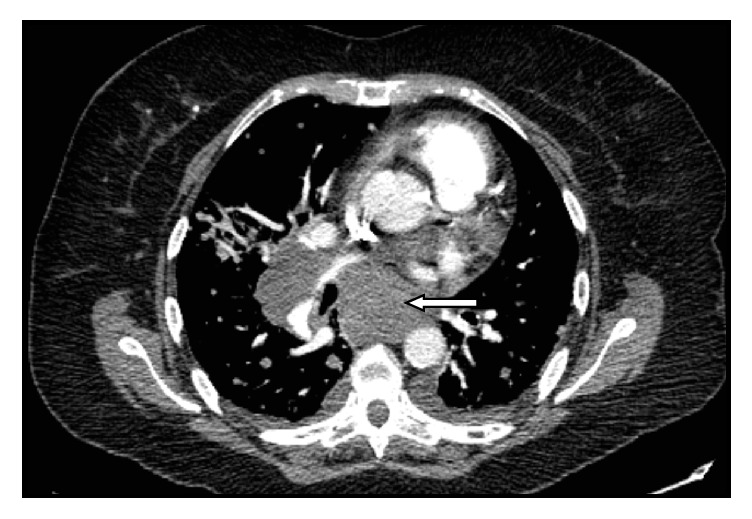
Preoperative computed tomography (CT) scan. This CT demonstrates large mediastinal and hilar lymph nodes with the largest being located in station VII (subcarinal nodes) with a 5.6 cm diameter (arrow) resulting in a mass effect on the bronchus and hilar vessels.

A 69-year-old female patient was diagnosed with stage IV C follicular thyroid carcinoma (pT3pN1bM1 20/21) with concomitant lung and mediastinal lymph node metastasis. Her medical history included restrictive lung disease associated with obesity (Body Mass Index = 42 Kg/m2) and hypertension. Preoperative thoracic and abdominal computed tomography scan (CT scan) demonstrated large mediastinal and hilar lymph nodes exerting a mass effect on the bronchus and hilar vessels, especially in the right lower lobe (Figure 1). The patient was treated with total thyroidectomy, radical neck dissection, and mediastinal lymph node resection by right posterolateral thoracotomy, followed by postoperative radioiodine ablation. Posttherapeutic iodine-131 whole-body scintigraphy combined with single-photon emission computed tomography/computed tomography (I^131^-SPECT/CT) showed an additional osteolytic lesion of the right iliac bone.

**Figure 2:**
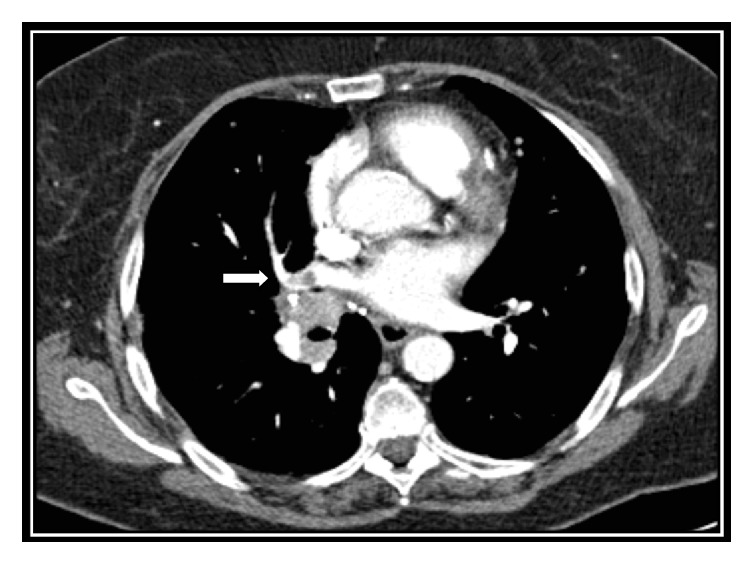
PET-FDG computed tomography scan performed 6 months after surgery and radioiodone ablation. The right superior pulmonary vein appears occluded by a thrombus (arrow).

Positron emission and computed tomography (PET-CT) scan performed 6 months later revealed stability of the multiple lung lesions. Nevertheless, the right superior pulmonary vein appeared occluded by a thrombus and in close contact with a hypermetabolic (SUV max 9.5) hilar mass with a 5 cm diameter (Figure 2). The patient was clinically asymptomatic and physical examination was unremarkable.

Anticoagulation with dalteparin 200 U/Kg once a day for 1 month was started followed by dalteparin 150 U/Kg daily for another 6 months. At six months' follow-up, the patient was still asymptomatic. She did not experience any other thrombotic event or embolic or bleeding complications. PET-CT scan revealed stability of the right pulmonary vein thrombosis as well as of the lung and mediastinal lymph node metastasis.

## 3. Discussion

Pulmonary vein thrombosis is a rare and potential life-threatening clinical entity that mainly occurs after thoracic surgery and lung transplantation [5]. It has also been associated with extracorporeal membrane oxygenator (ECMO) assistance [7], radiofrequency ablation for atrial fibrillation [9], thoracic tumors [6], and blunt chest trauma [10]. In PVT occurring after thoracic surgery or lung transplantation, the thrombus formation is believed to be promoted by blood flow stasis, endothelium damage, and activation of the thrombotic cascade [2, 3, 11–13]. In cases associated with thoracic tumors, the underlying mechanism seems to be related to the mechanical compression of the vein as well as the prothrombotic state associated with the malignancy itself [6]. Mechanical compression could be a sufficient cause for PVT, as outlined by a case resulting from a large hiatal hernia [14]. Several idiopathic cases of PVT have also been described where the underlying mechanism is more difficult to understand and could involve a systemic or local hypercoagulable state [1, 8, 15–17].

Clinical presentation of PVT is nonspecific. Following lung transplantation, PVT often presents in the early postoperative period with fever, dyspnoea, signs of pulmonary hypertension, and pulmonary oedema and it can be life-threatening [5, 10, 13]. Symptoms of idiopathic or secondary PVT can vary from purely respiratory (dyspnoea, haemoptysis, chest pain, and cough) [1, 8, 15, 17], to symptoms related to the complications themselves. Complications can be classified into two categories, those related to local impact of the thrombus with pulmonary oedema, infarction, right ventricular failure, or even lung gangrene [7, 13], and those resulting from peripheral embolic phenomena to the brain (embolic stroke, transient ischemic attack) [2, 4, 5, 18, 19], spleen [2], kidney [2, 14], or limb circulation [2, 16].

Diagnosis can be challenging especially in cases of idiopathic PVT. Contrast enhanced CT angiography scan is very precise for thrombus localisation [8, 14, 20] but cardiac-gated magnetic resonance imaging has also been reported to be accurate [6]. Transoesophageal echocardiography has been used for the diagnosis of PVT in the posttransplantation setting, and in idiopathic PVT cases as well with very good results [4, 5, 19].

Management of PVT depends on the underlying cause, when it can be identified. After thoracic surgery or transplantation, thrombolysis, surgical thrombectomy, or anastomotic revision is often suggested as the first-line treatment, depending on the size of the thrombus [11, 13]. In other settings, anticoagulation is the mainstay therapy, in order to prevent local extension of the thrombus and embolic spread. However, the duration of anticoagulation lacks evidence [3, 8, 14, 15]. Recently, a case of complete thrombus resolution after 3 months of treatment with dabigatran in a patient with idiopathic left upper pulmonary vein thrombosis has been reported [20]. Pulmonary lobectomy has also been performed in severe cases in order to treat or prevent lung gangrene [1, 17].

In our case, the different treatment options were discussed during a multidisciplinary team meeting that included pulmonologists, oncologists, cardiologists, and endocrinologists. The aim was to choose the optimal management for this asymptomatic patient suffering an advanced malignancy. Interventional treatments (thrombolysis, thrombectomy) were excluded and anticoagulation alone with low-molecular weight heparin was preferred with the objective of preventing local extension of the thrombus, as well as embolic phenomena; emphasis was given on close clinical follow-up.

## 4. Conclusions

PVT associated with metastatic thyroid cancer is rare, and literature reports are scarce. Risk factors include mechanical compression from the large mediastinal-hilar metastasis, the right posterolateral thoracotomy for lymph node resection, and the prothrombotic state associated with the malignancy itself. Management strategies with anticoagulation regimens in an asymptomatic patient mostly target the prevention of thrombus expansion as well as embolic phenomena.

## Abbreviations

CT scan: Computed tomography scan
I^131^-SPECT/CT: Iodine-131 whole-body scintigraphy with single-photon emission computed tomography/computed tomography
PET-CT: Positron emission and computed tomography
PVT: Pulmonary vein thrombosis.
